# Peroxisome Proliferator-Activated Receptors in Diabetic Nephropathy

**DOI:** 10.1155/2008/879523

**Published:** 2009-03-04

**Authors:** Shinji Kume, Takashi Uzu, Keiji Isshiki, Daisuke Koya

**Affiliations:** ^1^Department of Medicine, Shiga University of Medical Science, Otsu, Shiga 520-2192, Japan; ^2^Division of Endocrinology and Metabolism, Department of Internal Medicine, Kanazawa Medical University, Ishikawa 920-0293, Japan

## Abstract

Diabetic nephropathy is a leading cause of end-stage renal disease, which is increasing in incidence worldwide, despite intensive treatment approaches such as glycemic and blood pressure control in patients with diabetes mellitus. New therapeutic strategies are needed to prevent the onset of diabetic nephropathy. Peroxisome proliferator-activated receptors (PPARs) are ligand-activated nuclear transcription factors that play important roles in lipid and glucose homeostases. These agents might prevent the progression of diabetic nephropathy, since PPAR agonists improve dyslipidemia and insulin resistance. Furthermore, data from murine models suggest that PPAR agonists also have independent renoprotective effects by suppressing inflammation, oxidative stress, lipotoxicity, and activation of the renin-angiotensin system. This review summarizes data from clinical and experimental studies regarding the relationship between PPARs and diabetic nephropathy. The therapeutic potential of PPAR agonists in the treatment of diabetic nephropathy is also discussed.

## 1. INTRODUCTION

The incidence and prevalence of type 2 diabetes mellitus (DM) have been increasing worldwide since the 1980s, and this rise is estimated to continue in the future [1, 2]. Diabetic nephropathy is a common complication of DM and represents one of the major challenges for modern nephrology as the most common cause of end-stage renal disease, accounting for about 40% of new cases [3, 4]. The increasing prevalence of DM and its complications including diabetic nephropathy have therefore become a major health problem worldwide, and new therapeutic strategies to prevent diabetic nephropathy are urgently needed.

Peroxisome proliferator-activated receptors (PPARs) are ligand-activated transcription factors belonging to the nuclear hormone receptor superfamily. They were originally cloned from rodent liver while screening for molecular mediators of peroxisome proliferation [5, 6]. Three isoforms have been cloned (PPAR*α*, PPAR*β*/*δ*, and PPAR*γ*) and characterized. Each has a unique expression pattern and ligand-binding specificity, as well as distinct metabolic functions [7]. PPARs regulate diverse cell functions, including fatty acid metabolism, adipocyte differentiation, inflammation, atherosclerosis, and cell cycle [8–11]. PPAR*α* plays an important role in lipid metabolism in several tissues including liver and kidney [12]. PPAR*β*/*δ* is associated with cell survival and colon carcinogenesis [13] and was recently implicated as an important regulator of mitochondrial biogenesis and subsequent lipid metabolism in skeletal muscle [14]. PPAR*γ* plays a pivotal role in adipogenesis, and its activation by thiazolidinediones (TZDs) improves insulin sensitivity via this role in adipocyte differentiation [15]. Accordingly, TZDs are widely used as oral antidiabetic agents in patients with type 2 diabetes [15, 16]. It is clear that substantial experimental and clinical research is still needed to clarify the role of PPAR*γ* in the whole body physiology and the pathophysiology of various diseases such as diabetes, obesity, hypertension, atherosclerosis, and cancer.

In addition to the demonstrated physiological roles, several clinical and experimental studies have implicated PPARs in the pathogenesis of diabetic nephropathy. This review summarizes these clinical and experimental data with a particular focus on the therapeutic potential of PPAR modulators in diabetic nephropathy.

## 2. STRUCTURE OF PPARs

**Figure 1:**
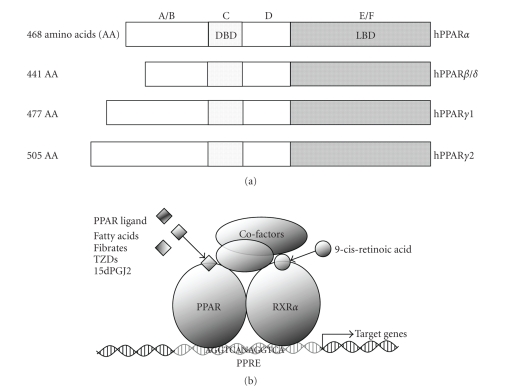
Structure and action of PPARs. (a) Domain structure of human PPARs. (b) Molecular mechanism of PPARs. After ligand binding, PPARs undergo conformational change with RXR and cofactors.

PPAR was initially identified in a mouse cDNA library in 1990 [6], and since then three PPARs have been cloned: PPAR*α*, PPAR*β*/*δ*, and PPAR*γ* (Figure 1(a)) [7]. PPAR*γ* mRNA has three splicing forms derived from a single gene in human [17]. There are no splicing variants of PPAR*α* or PPAR*β*/*δ* mRNA. Two PPAR*γ* protein isoforms result from the translation of each of the three PPAR*γ* mRNAs to produce PPAR*γ*1 and *γ*2 [18], with both PPAR*γ*1 and PPAR*γ*3 mRNAs giving rise to the same protein, PPAR*γ*1. PPAR*γ*2 is the larger of the two isoforms, with 30 additional N-terminal amino acids. Due to different promoter usage, PPAR*γ*1 and PPAR*γ*2 have different expression patterns [19].

All PPARs possess four domains similar to those found in other nuclear hormone receptors [5, 20]: an NH2-terminal ligand-independent transactivation domain (activation function-1 (AF-1)), which regulates PPAR activity (A/B domain) [21, 22]; a DNA-binding domain of 70 amino acids (two zinc fingers) (DBD, C domain); a docking domain for cofactors (D domain); a COOH-terminal region containing the ligand-binding domain (LBD) and AF-2 domain (E/F domain). DBD and LBD are approximately 70% homologous among the three PPARs.

## 3. PPAR LIGANDS

PPARs are ligand-activated transcriptional factors belonging to the nuclear hormone receptor superfamily, whereby modulation of target gene transcription depends on the binding of ligands to the receptor. PPARs form heterodimers with the 9-cis retinoic acid receptor, retinoid X receptor (RXR*α*). Activation of the PPAR:RXR*α* heterodimers by PPAR ligands and/or RXR ligands triggers a conformational change in the receptors. This in turn allows the heterodimers to bind to PPAR responsible element containing the sequence AGGTCANAGGTCA in the promoter region of the target genes, and thus modulate gene transcription (Figure 1(b)).

Many ligands including natural and synthetic compounds have been identified for each PPAR isoform in both functional (cell-based transactivation efficiency) and in vitro interaction assays [8, 23]. The different amino acids sequences in the LBD of each PPAR provide the molecular basis for ligand specificity. Each PPAR can accommodate several structurally diverse ligands due to a large ligand-binding pocket [24]. PPAR*α* binds unsaturated fatty acids with the highest affinity of the three isoforms [25–28]. Natural ligands for PPAR*γ* also include several unsaturated fatty acids such as oleate, linoleate, eicosapentaenori and arachidonic acids, and 15dPGJ2 [8, 23, 29, 30]. TZD compounds such as troglitazone (was the first agent of this class on the market, but withdrawn due to liver toxicity), ciglitazone, pioglitazone, and rosiglitazone act as synthetic PPAR*γ* ligands and promote adipocyte differentiation via activation of the receptor [23, 31–35]. Termisaltan, an angiotensin II type 1 receptor blocker (ARB), was recently shown to bind PPAR*γ* and reduce blood glucose levels [36, 37].

## 4. DISTRIBUTION OF PPARs IN KIDNEY

Expression of the three PPAR isoforms has been examined in many species including Xenopus, rat, mouse, rabbit, and human. PPAR*α* is mainly expressed in tissues exhibiting high catabolic rates of fatty acids such as adipose tissue, liver, heart, and skeletal muscle [38, 39]. PPAR*β*/*δ* is ubiquitously expressed, while PPAR*γ* is highly expressed in white and brown adipose tissues that store large amounts of fatty acids, and in other selected tissues at low levels such as heart, liver, immune cells (monocytes and macrophages), placenta, and colon [40–42].

All three PPARs are expressed in the kidney [38, 41–43]. PPAR*γ* mRNA has been demonstrated in the medullary collecting ducts and pelvic urothelium of kidney [44], as well as in isolated glomeruli and cultured mesangial cells [45, 46]. PPAR*α* and *γ*1, but not *γ*2, protein was detected in kidney tissue by immunoblot analysis, while immunohistochemical analysis revealed PPAR*α* and *γ*1 proteins in the nuclei of mesangial cells and epithelial cells in glomeruli, proximal and distal tubules, the loop of Henle, medullary collecting ducts, and the intima/media of renal vasculatures [47]. Large amounts of PPAR*α* have also been detected in proximal tubular cells, and renal lipid metabolism is highly regulated by PPAR*α* [48]. In contrast to PPAR*α*, PPAR*γ* protein is highly expressed in the nephron segment, predominantly in collecting ducts, implicating PPAR*γ* in systemic water and sodium retention [49, 50].

## 5. EXPERIMENTAL (ANIMAL) STUDIES

PPAR*γ* is the best characterized of the PPAR isoforms in diabetic animal models. The first evidence for a possible renoprotective effect of PPAR*γ* agonists came 15 years ago, with the TZD compound troglitazone decreasing urinary albumin excretion and reducing blood pressure in obese Zucker rats [51]. Further studies since then also showed the beneficial effects of TZD compounds on renal injury in type 1 and type 2 diabetic animal models, as summarized in Table 1 [50, 52–60]. Several experimental studies also showed similar or superior protection against diabetic nephropathy for PPAR*γ* agonists such as TZD, with results comparable to other renoprotective agents such as renin-angiotensin system blockers.

PPAR*α* is highly expressed in renal proximal tubules and helps to maintain a sustained balance of energy production and expenditure in the kidney [61]. The role of PPAR*α* in renal cortex lipid metabolism was demonstrated when the activation of PPAR*α* by clofibrates induced expression of *β*-oxidation enzymes [62]. In *db*/*db* type 2 diabetic mice [63] and Zucker diabetic rats [64], treatment with PPAR*α* activator, fenofibrate, improved urinary albumin excretion rates and glomerular mesangial expansion. These experimental studies suggest PPAR*α* agonists as potentially useful therapeutic agents for diabetic nephropathy.

## 6. HUMAN CLINICAL TRIALS

Several clinical trials of PPAR*γ* agonists have been conducted over the past decade that together confirm the renoprotective action of PPAR*γ* (Table 2) [65–79]. PPAR*γ* agonist, TZD, is an approved therapeutic agent for glycemic control in patients with type 2 DM, and thus is effective in preventing type 2 diabetic nephropathy. The beneficial effect of pioglitazone on urinary albumin excretion was also demonstrated in large, multicenter intervention studies, which compared the general efficacy and safety of TZD agents to other oral antidiabetic agents in patients with type 2 DM over 1 year. Either pioglitazone or the antidiabetic, metformin, was given to 639-randomized patients already receiving a sulfonylurea [72]. Although the two regimens had comparable effects on glycemic control, urinary albumin excretion was reduced by 15% in the group receiving pioglitazone and increased by 2% in the metformin group. In another study from the same group on drug-naive patients with type 2 DM, pioglitazone significantly reduced urinary albumin excretion, whereas metformin had no effect. A similar follow-up study showed that administration of pioglitazone in those patients who had previously received metformin therapy was associated with a decreased urinary albumin excretion of 10%, whereas another TZD compound, gliclazide, caused an increase of 6% [74]. Taken together, these data from both large and small clinical studies showed that PPAR*γ* agonists have a beneficial effect on diabetic nephropathy compared to other antidiabetic agents.

It should be noted that PPAR*γ* agonists could potentially cause heart failure due to the associated water retention. Recent clinical trials in patients with impaired glucose tolerance (IGT) and/or impaired fasting glucose (IFG) showed that rosiglitazone, which reduces the onset of diabetes, also reduced the development of renal disease; however, it increased the adverse risk of heart failure, compared to ramipril [79]. Therefore, PPAR*γ* agonists should be used only with intensive monitoring of volume retention in patients with cardiac risk factors.

Clinical evidence also suggests the beneficial effect of PPAR*α* ligands on diabetic nephropathy. Treatment of type 2 diabetes-associated dyslipidemia with gemfibrozil, an antidyslipidemic agent and PPAR*α* activator, stabilized urinary albumin excretion rates [80, 81]. In addition, a large randomized controlled trial in 2005 determined that long-term fenofibrate therapy significantly reduced the rate of progression to albuminuria in patients with type 2 DM [82]. Although not extensive, these clinical data suggest the therapeutic efficacy of PPAR*γ* agonists in preventing diabetic nephropathy.

### 6.1. Effects of PPAR*γ* ligands on diabetic nephropathy

#### 6.1.1. Improving hyperglycemia

The Diabetes Control and Complications Trial (DCCT) and the United Kingdom Prospective Diabetes Study (UKPDS) suggested that the adverse effects of hyperglycemia on metabolic pathways are the main causes of long-term complications such as kidney disease in diabetes [83, 84]. TZDs are a new class of oral antidiabetic agents used widely to improve insulin resistance, hyperinsulinemia, and hyperglycemia in patients with type 2 diabetes [85–87]. Since the improvement of hyperglycemia in such patients can prevent the development and progression of diabetic nephropathy, TZDs are potential protective agents for nephropathy in type 2 diabetes patients and animal models by virtue of their insulin-sensitizing action [66].

#### 6.1.2. Lowering blood pressure with or without improved insulin resistance

Hypertension is commonly linked to obesity and insulin resistance [88]. TZDs have a possible antihypertensive effect through improvement of insulin resistance because insulin sensitivity is related to blood pressure levels both in diabetic animals and patients [50, 89–92]. On the other hand, PPAR*γ* ligands could directly affect vascular function because of their expression in endothelial cells and vascular smooth muscle cells (VSMCs) [93–95]. Indeed, pioglitazone lowered the blood pressure in 5/6 nephrectomized hypertensive rats, and the effect was not associated with insulin resistance [96, 97]. The demonstrated antihypertensive effects of TZDs could involve the release of vasodilators such as nitric oxide and prostaglandins [98], the decrease in fatty acid levels, and/or modification of vasoactive peptide synthesis including endothelin-1 [47]. Recently, PPAR*γ* downregulated the expression of angiotensin II type 1 receptor and in turn decreased vascular smooth muscle tone, thereby reducing vascular contractility [99]. Although the underlying functional mechanisms remain unclear, PPAR*γ* expression probably contributes to blood pressure regulation through multiple mechanisms.

### 6.2. Renoprotective effects of PPAR*γ* ligands due to mechanisms other than changes in blood glucose levels

TZD treatment ameliorated renal abnormalities in streptozotocin- (STZ-) induced diabetic rats, a type 1 diabetic model, without changing blood glucose levels [54, 56]. These findings suggest that the protective effects of PPAR*γ* ligands on diabetes-induced renal dysfunction are independent of its insulin-sensitizing property. Multiple biochemical mechanisms have been proposed to explain the adverse effects of hyperglycemia in diabetes, and the effects of PPAR*γ* ligands on each of these mechanisms is discussed below.

#### 6.2.1. Amelioration of DGK-DAG-PKC pathway activation

The diacylglycerol- (DAG-) protein kinase C- (PKC-) extracellular signal-regulated kinase (ERK) pathway is enhanced in mesangial cells cultured under high-glucose conditions and in glomeruli isolated from streptozotocin- (STZ-) induced diabetic rats [100–103]. In these animals, troglitazone ameliorated the diabetes-associated increases in glomerular filtration rate, urinary albumin excretion, and mRNA expressions of extracellular matrix (ECM) proteins (fibronectin and type IV collagen) and transforming growth factor-*β* (TGF-*β*) without changing the blood glucose levels [56]. These findings provided the first evidence that PPAR*γ* ligands can protect glomerular function independent of their insulin-sensitizing action. In mesangial cells cultured under high-glucose conditions and in isolated glomeruli from diabetic rats, it was confirmed that TZDs inhibited the accumulation of DAG and its subsequent activation of the PKC-ERK pathway. Furthermore, another TZD, pioglitazone, also prevented DAG-PKC-ERK pathway upregulation in mesangial cells exposed to high glucose [56]. Finally, TZDs and potent PPAR*γ* ligand, 15dPGJ2, increased the protein expression of DGK to block DAG-PKC signaling in endothelial cells [103].

#### 6.2.2. Attenuation of oxidative stress

Increased oxidative stress is observed in renal glomeruli and a variety of vascular and nonvascular tissues exposed to hyperglycemia [104–106]. Troglitazone has potent antioxidant effects, evident by it suppressing phosphoenolpyruvate gene expression in vitro and scavenging reactive oxygen species in vivo [107]. It also normalizes the decrease in plasma lipid hydroperoxide concentration and increase of superoxide dismutase activity in Otsuka Long-Evans Tokushima Fatty rats, a type 2 diabetic animal model, and improves the decreased skin blood flow in STZ-induced diabetic rats [98, 108, 109]. Pioglitazone also reduces oxidative stress in the kidney of alloxan-induced diabetic rabbits [110, 111] and reduces renal lipid peroxides, urinary isoprostane excretion, and expression of p47 *phox* and gp91 *phox* in high-fat diet-induced obese rats [112].

#### 6.2.3. Suppression of inflammation

Hyperglycemia and the diabetic state can induce cytokine production in some tissues. In diabetic nephropathy, macrophages infiltrates appear in glomeruli and the interstitial spaces between tubules [113, 114]. Both PPAR*α* and *γ* have potent anti-inflammatory effects in macrophages [115, 116]. The endogenous and potent PPAR*γ* ligand, 15dPGJ2, is a natural metabolite derived from prostaglandin (PG)D2, the most abundant prostaglandin in normal tissues with the highest binding affinity to PPAR*γ* of the J-series prostaglandins [117]. Several studies demonstrated that the anti-inflammatory effect of 15dPGJ2 or TZDs seems to be regulated through transcriptional inhibition by both PPAR*γ*-dependent [115, 116, 118] and PPAR*γ*-independent mechanisms [119–121]. Nuclear factor-*κ*B (NF-*κ*B), a well-known inflammatory transcription factor, is repressed by 15dPGJ2 in a PPAR*γ*-independent manner [122]. It was also reported that 15dPGJ2 inhibits interleukin-1*β*- (IL-1*β*-) induced cyclooxygenase-2 expression and PGE2 production independently of PPAR*γ* activation in mesangial cells, by suppressing ERK and c-Jun NH2-terminal kinase (JNK) pathways and AP-1 activation [123]. Another TZD agent, ciglitazone, inhibited platelet-derived growth factor-induced mesangial cell proliferation without changing ERK activation, through inhibiting the activation of serum response element directly [124].

#### 6.2.4. Modification of atherosclerotic changes

Renal atherosclerotic changes such as renovascular stenosis and atheroemboli are common findings in elderly diabetic patients and are known to accelerate renal dysfunction [125, 126]. PPAR*γ* activation also may modify the progression of atherosclerosis through multiple mechanisms including foam cell differentiation, inflammatory reactions, and cell proliferation [127]. The infiltrating monocytes take up oxidized low-density lipoprotein (OxLDL) via scavenger receptors, resulting in the accumulation of intracellular lipids and generation of foam cells [127]. The OxLDL scavenger receptor, CD36, is under direct control of PPAR*γ* [29, 30]. OxLDLs include natural PPAR*γ* agonists such as 9-hydroxyoctadecadienoic acid (HODE) and 13-HODE. Furthermore, OxLDL induces the expression of PPAR*γ* [115], which has an anti-inflammatory effect in monocytes by reducing proinflammatory cytokine production [115] via inhibition of proinflammatory transcription factors such as NF*κ*B, AP-1, and STATs [116]. PPAR*γ* has other effects on atherosclerosis including induction of apoptosis in monocytes [128], inhibition of VSMC proliferation [94, 129], and suppression of matrix metalloproteinase-9 expression [130].

### 6.3. Effects of PPAR*γ* ligands in tubular tissue

Patients with diabetic nephropathy frequently show a nephrotic state, whereby large quantities of albumin enter the renal tubular system and carry with it a heavy load of fatty acids. Albumin-bound fatty acids can activate PPAR*γ* and induce apoptosis of proximal tubular cells. PPAR*γ* agonists might inhibit tubular cell proliferation, whereas activation of albumin-bound fatty acids is accompanied by increased proliferation [131]. In particular, pioglitazone increases the tubular cell albumin uptake and reverses the expression of inflammatory and profibrotic markers, monocyte chemoattractant protein-1 (MCP-1) and TGF-*β* [132].

## 7. INVOLVEMENT OF PPAR*α* AND PPAR*β*/*δ* IN DIABETIC NEPHROPATHY

PPAR*α* agonists have renoprotective effects as mentioned above. One possible mechanism underlying PPAR*α* action on mesangial matrix production may be related to hyperglycemia or TGF*β* signaling [133]. Clofibrate directly inhibited oxidative stress-induced TGF*β* expression in mesangial cells [133], while fenofibrate downregulated TGF*β* and TGF*β* receptors type II expression and decreased type IV collagen accumulation in diabetic glomeruli, and inhibited the production of PAI-1 in diabetic animals [63, 64].

PPAR*β*/*δ* is expressed equally in the renal cortex and medulla, although the role of PPAR*β*/*δ* in the kidney remains poorly understood [41]. Overexpression of this isoform protected cultured medullary interstitial cells from hypertonicity-induced cell death, suggesting that PPAR*β*/*δ* is an important survival factor under hypertonic conditions in renal medulla [134]. However, there are no reports regarding the effect of PPAR*β*/*δ* on diabetic nephropathy. Further evidence from both clinical and experimental studies is necessary to clarify the therapeutic potential of PPAR*β*/*δ* and PPAR*α* agonists in diabetic nephropathy.

Several recent studies suggested lipotoxicity from renal lipid accumulation as a possible pathogenic mechanism underlying certain forms of renal injury including diabetic nephropathy [135–137]. PPAR*α* regulates lipid metabolism in the kidney [48], and PPAR*α* knockout mice develop severe interstitial lesions induced by fatty acid overload [138]. PPAR*α* agonists may, therefore, decrease lipotoxicity and, consequently, inhibit the progression of diabetic nephropathy. PPAR*β*/*δ* also regulates lipid metabolism and particularly lipid oxidation in several tissues, although its exact roles in the kidney remain unclear. Thus, both PPAR*β*/*δ* and PPAR*α* agonists could be implemented in new therapeutic strategies designed to prevent diabetic nephropathy by reducing renal lipotoxicity. Further studies are required to prove this possibility.

## 8. CONCLUSION AND PERSPECTIVES

The increased incidence of diabetic nephropathy has become a major health problem worldwide. As discussed in this review, PPARs comprise a subfamily of nuclear receptors and transcription factors that play critical roles in modulating insulin resistance, hypertension, dyslipidemia, obesity, hypertension, and inflammation. Given the close relationship between PPAR activity and these metabolic alterations, PPAR agonists are promising therapeutic agents for diseases including type 2 diabetes, obesity, hypertension, hyperlipidemia, and atherosclerosis. Fibrate PPAR*α* agonists and TZD PPAR*γ* agonists are already used successfully as clinically effective hypolipidemic drugs and insulin sensitizers. PPAR*β*/*δ* agonists may provide additional insulin and lipid modulators via their effects on skeletal muscle. In addition, there is an increasing evidence suggesting that all three PPARs contribute to the metabolic control of renal function and are involved in the pathogenesis of diabetic nephropathy. PPAR*γ* agonists are available as optional therapeutic agents for nephropathy in type 2 diabetes. In the near future, both PPAR*α* and PPAR*β*/*δ* agonists might be added to that strategy with further evidence that these agents have a proven renoprotective effect in diabetic animals and patients.

## Figures and Tables

**Table 1:** Animal studies.

Authors	TZD	Animal model	Duration	Effect on UAE	Effect on BP	Other effects
Model of type 1 diabetes						

Fujii et al. [54]	Tro	STZ-induced diabetic rats	12 weeks	↓	NS	ND
Isshiki et al. [56]	Tro	STZ-induced diabetic rats	12 weeks	↓	ND	Hyperfiltration ↓
Nicholas et al. [58]	Tro	STZ-induced diabetic rats	12 weeks	↓	NS	ND
Yamashita et al. [60]	Tro, pio	STZ-induced diabetic SHR rats	12 weeks	↓	NS	Loss of glomerular basement membranes ↓

Model of type 2 diabetes						

Yoshioka et al. [51]	Tro	Obese Zucker rats	4 and 8 weeks	↓	↓	ND
Fujiwara et al. [55]	Tro	Wistar fatty rats	24 weeks	↓	↓	ND
Yoshimoto et al. [50]	Pio	Diabetic Wistar fatty rats	13 weeks	↓	↓	Glomerulosclerosis ↓ intrarenal arteriolosclerosis ↓
Tanimoto et al. [59]	Pio	Diabetic KK/Ta mice	4 and 8 weeks	↓	NS	Glomerular enlargement ↓
Buckingham et al. [53]	Rosi	Obese Zucker rats	4 and 9 months	↓	↓	Glomerulosclerosis ↓ tubulointerstitial fibrosis ↓
Baylis et al. [52]	Rosi	Obese Zucker rats	6 months	↓	NS	Glomerulosclerosis ↓ tubulointerstitial fibrosis ↓
Khan et al. [57]	Rosi	Obese Zucker rats	12 weeks	↓	↓	ND

TZD, thiazolidinedione; Tro, troglitazone; Pio, pioglitazone; Rosi, rosiglitazone; STZ, streptozotocin; SHR, spontaneously hypertensive rats; UAE, urinary albumin excretion; BP, blood pressure; NS, no significant effects; ND: not determined; ↓, significant reductions.

**Table 2:** Human clinical studies.

Authors	subjects (Type 2 DM)	*n*	regimens	Duration	Effect on UAE (%)	Effect on BP (mmHg)
Sironi et al. [65]	hyp	40	200 mg toroglitazone versus plb	8 weeks	+11%	−4/−3

Imano et al. [66]	mA, hyp	30	400 mg toroglitazone versus 500 mg metformin	12 weeks	−39%^a^	−3/0

Nakamura et al. [67]	mA or MA	32	400 mg toroglitazone versus 5 mg glibenclamide	12 months	−67%^a^in mA 0% in MA	−6^c^

Nakamura et al. [68]	mA	45	30 mg Pio versus 5 mg glibenclamide versus 0.6 mg Vog	3 months	−66%^a^	−6/−4

Nakamura et al. [69]	mA	28	30 mg Pio versus plb	6 months	−59%^a^	−4^c^

Aljabri et al. [70]	mA, hyp	62	30–45 mg Pio versus isophane insulin	16 weeks	−44%	−8/−5

Yanagawa et al. [71]	mA, hyp	40	Pio versus Met or glibenclamide	12 weeks	−45%^a^	NA

Hanefeld et al. [72]	mA, hyp	639	15–45 mg Pio versus 850–2550 mg metformin	12 months	−15%^a^	NA

Schernthaner et al. [73]	hyp	1199	15–45 mg Pio versus 850–2550 mg metformin	12 months	−19%^a^	NA

Matthews et al. [74]	hyp	630	15–45 mg Pio versus 80–320 mg glibenclamide	12 months	−10%^a^	NA

Agarwal et al. [75]	MA, hyp	44	Pio versus Glip	4 months	−7%	+3.7/+2.2

Lebovitz et al. [76]	mA, hyp	493	4 or 8 mg Rosi versus plb	26 weeks	4 mg group: −14% 8 mg group: −22%^a^	NA

Sarafidis et al. [77]	hyp, mA	20	4 mg Rosi	6 months	−35%^a^	−5.4^a^/−4.1^a^

Pistrosch et al. [78]	mA, hyp	19	non-mA patients: Rosi versus Nat, mA patients: Rosi versus plb	12 weeks	non-mA patients: +18%^b^, mA patients: −66%^a,b^,	NA

^a^Significant changes from baseline levels or other groups; ^b^change versus the group compared; ^c^mean change for systolic BP versus baseline in patients treated with the TZD. DM, diabetes mellitus; hyp, hypertension; mA, microalbuminuria; MA, macroalbuminuria; Glip, glipizide; Nat, nateglinide; plb, placebo; Pio, pioglitazone; Vog, voglibose; UAE, urine albumin excretion; NA, changes in blood pressure levels not applicable.
